# Articles from the 15th ICI Meeting in Milan, Italy

**DOI:** 10.3389/fimmu.2014.00356

**Published:** 2014-07-28

**Authors:** Kendall A. Smith

**Affiliations:** ^1^Division of Immunology, Department of Medicine, Weill Medical College, Cornell University, New York, NY, USA

**Keywords:** ICI2013, immunology, IUIS, Milan, SIICA

The First International Congress in Immunology was held in 1971 in Washington, DC, USA. Since that initial gathering of worldwide immunologists, we have evolved considerably as the articles selected from presentations at the 15th ICI and compiled here attest. By 1971, the two main systems involved in adaptive immunity had been described and the two major types of lymphocytes, B cells and T cells, recognized by their surface expression of immunoglobulin molecules and theta antigen had just been described. Years later, I was riding in a bus at an immunology meeting that was headed to the conference banquet. My companion in the seat next to me happened to mention that he was present at the first ICI, and I replied that I was also there. My seatmate then went on to say that everyone that he spoke to at that inaugural meeting felt that the major mysteries in immunology had been uncovered and now we just needed to dot the I’s and cross the T’s. Needless to say that we have all come a long way in the past 42 years, and great strides have been made in delineating the characteristics of the immune system, as exemplified here in this compilation of contributions stemming from the 15th ICI.

## Conflict of Interest Statement

The author declares that the research was conducted in the absence of any commercial or financial relationships that could be construed as a potential conflict of interest.

